# Standardisierte und qualitätsgesicherte prädiktive PD-L1-Testung im oberen Gastrointestinaltrakt

**DOI:** 10.1007/s00292-023-01215-3

**Published:** 2024-01-03

**Authors:** G. Baretton, F. Lordick, Die Interdisziplinäre Expertengruppe, T. Gaiser, R. Hofheinz, D. Horst, S. Lorenzen, M. Möhler, C. Röcken, P. Schirmacher, M. Stahl, P. Thuss-Patience, K. Tiemann

**Affiliations:** 1 Institut für Pathologie, Universitätsklinikum Carl Gustav Carus, TU Dresden, Fetscherstr. 74, 01307 Dresden, Deutschland; 2 Medizinische Klinik II (Onkologie, Gastroenterologie, Hepatologie und Pneumologie) und Universitäres Krebszentrum Leipzig, Universitätsmedizin Leipzig, Leipzig, Deutschland; 3 PATHOLOGIE SPEYER Gemeinschaftspraxis GbR, Speyer, Deutschland; 4 Universitätsmedizin Mannheim, Mannheim, Deutschland; 5 Institut für Pathologie, Charité – Universitätsmedizin Berlin, Berlin, Deutschland; 6 III. Medizinische Klinik, Klinikum rechts der Isar, München, Deutschland; 7 I. Medizinische Klinik und Poliklinik, Universitätsmedizin Mainz, Mainz, Deutschland; 8 Institut für Pathologie, Christian-Albrechts-Universität, Kiel, Deutschland; 9 Pathologisches Institut, Universitätsklinikum Heidelberg, Heidelberg, Deutschland; 10 Klinik für Internistische Onkologie & Onkologische Palliativmedizin, KEM | Evang. Kliniken Essen-Mitte, Evang. Huyssens-Stiftung Essen-Huttrop, Essen, Deutschland; 11 Charité Centrum Tumormedizin CC14, Campus Virchow-Klinikum, Charité – Universitätsmedizin Berlin, Berlin, Deutschland; 12 Institut für Hämatopathologie, Hamburg, Deutschland

**Keywords:** PD-L1-Testung, Immuncheckpointinhibitor, Prädiktive Biomarker, Adenokarzinom des gastroösophagealen Übergangs, Adenokarzinom des Magens, Plattenepithelkarzinom des Ösophagus, PD-L1 testing, Immune checkpoint inhibitor, Predictive biomarkers, Gastroesophageal junction adenocarcinoma, Gastric adenocarcinoma, Esophageal squamous cell carcinoma

## Abstract

Infolge der hohen Zulassungsdynamik sowie der wachsenden Anzahl an immunonkologischen Therapiekonzepten nimmt die Komplexität der Therapieentscheidung und -steuerung im Bereich der Karzinome des Ösophagus, gastroösophagealen Übergangs und Magens stetig zu. Da die Indikationsstellung bei den derzeit in der Europäischen Union zugelassenen PD-1-Inhibitoren häufig an die Expression von PD-L1 (Programmed Cell Death Ligand 1) gekoppelt ist, ist die Bestimmung dieses gewebebasierten prädiktiven Markers durch die Pathologie für die Stratifizierung der Behandlung von maßgeblicher Bedeutung. Auch wenn die immunhistochemische Bestimmung des PD-L1-Expressionsstatus zu den am besten untersuchten, therapierelevanten Biomarkern für eine immunonkologische Behandlung gehört, ergeben sich aufgrund der hohen Heterogenität der Karzinome des oberen Gastrointestinaltrakts im klinisch-diagnostischen Alltag Herausforderungen in Bezug auf die Implementierung, Standardisierung und Interpretation der Testung. Eine interdisziplinäre Expertengruppe aus Deutschland hat zu relevanten Fragen aus dem klinisch-pathologischen Alltag Stellung bezogen, die das Ausgangsmaterial, die qualitätsgesicherte Testung und die Befundinterpretation betreffen und Empfehlungen für eine strukturierte Befunderstellung erarbeitet.

## Abstract (en)

As a result of the high approval dynamics and the growing number of immuno-oncological therapy concepts, the complexity of therapy decisions and control in the area of carcinomas of the esophagus, gastroesophageal junction and stomach is constantly increasing. Since the treatment indication for PD‑1 inhibitors that are currently approved in the European Union is often linked to the expression of PD-L1 (programmed cell death-ligand 1), the evaluation of tissue-based predictive markers by the pathologist is of crucial importance for treatment stratification. Even though the immunohistochemical analysis of the PD-L1 expression status is one of the best studied, therapy-relevant biomarkers for an immuno-oncological treatment, due to the high heterogeneity of carcinomas of the upper gastrointestinal tract, there are challenges in daily clinical diagnostic work with regard to implementation, standardization and interpretation of testing. An interdisciplinary group of experts from Germany has taken a position on relevant questions from daily pathological and clinical practice, which concern the starting material, quality-assured testing and the interpretation of pathological findings, and has developed recommendations for structured reporting.

Mit mehr als 1,5 Mio. neuen Fällen weltweit gehören Ösophagus- und Magenkarzinome zu den häufigsten malignen Tumoren [1]. Die Prognose fortgeschrittener oder metastasierter Malignome im oberen Gastrointestinaltrakt, zu denen Karzinome des Ösophagus, des gastroösophagealen Übergangs sowie des Magens gehören, ist ungünstig und das Überleben nach einer palliativ intendierten Standard-Chemotherapie beträgt in aktuellen klinischen Studien meist weniger als ein Jahr. Hinzu kommt, dass ein großer Teil der Karzinome häufig erst im fortgeschrittenen oder metastasierten und damit auch oft inoperablen Stadium diagnostiziert wird und die Patienten zu diesem Zeitpunkt oftmals nicht mehr mit kurativer Intention behandelbar sind.

Durch die Anwendung von Immuncheckpoint-Inhibitoren (ICI) haben sich die Therapiemöglichkeiten über die alleinige Kombinationschemotherapie hinaus erweitert. Aktuelle Phase-III-Studien haben das Potenzial einer Hinzunahme von ICI auch in der Erstliniensituation eines lokal fortgeschrittenen oder metastasierten Karzinoms im oberen Gastrointestinaltrakt aufgezeigt. Die Empfehlung zur Anwendung von ICI ist in den aktuellen internationalen Leitlinien implementiert [2–4].

In der Phase-III-Studie KEYNOTE-590 konnte bei Patienten mit Expression von PD-L1 (Combined Positive Score [CPS] ≥ 10) sowohl bei fortgeschrittenen Plattenepithelkarzinomen des Ösophagus als auch bei HER2(„human epidermal growth factor receptor 2“)-negativen Adenokarzinomen des Ösophagus und gastroösophagealen Übergangs ein signifikanter Vorteil im Gesamtüberleben nachgewiesen werden, wenn die Erstlinientherapie mit Pembrolizumab in Kombination mit Cisplatin und 5‑Fluorouracil (5-FU) vs. alleinige Chemotherapie erfolgte (13,9 vs. 8,8 Monate; Hazard Ratio [HR]: 0,57; *p* < 0,0001) [5].

In der dreiarmigen CheckMate-648-Studie, ebenfalls eine Phase-III-Studie, ergab sich für die Erstlinientherapie eines nicht resezierbaren fortgeschrittenen, rezidivierten oder metastasierten PD-L1-positiven Plattenepithelkarzinoms des Ösophagus (Tumor Proportion Score [TPS] ≥ 1 %) sowohl mit Nivolumab in Kombination mit Cisplatin und 5‑FU (15,4 vs. 9,1 Monate; HR: 0,54; *p* < 0,001) als auch mit der alleinigen ICI-Kombination aus Nivolumab plus Ipilimumab (13,7 vs. 9,1 Monate; HR: 0,64; *p* = 0,001) ein signifikanter Vorteil beim Gesamtüberleben gegenüber der alleinigen Chemotherapie [6]. In einer weiteren Erstlinientherapiestudie (CheckMate-649) konnte beim HER2-negativen Adenokarzinom des Magens, gastroösophagealen Übergangs bzw. Ösophagus ein signifikanter Vorteil der kombinierten Immunchemotherapie vs. alleinige Chemotherapie (FOLFOX oder CapeOx) beim primären Endpunkt Gesamtüberleben nachgewiesen werden: Bei PD-L1-positiven Tumoren (CPS ≥ 5) führte die Zugabe von Nivolumab zu einer medianen Verlängerung des Überlebens auf 14,4 vs. 11,1 Monate (HR 0,71; *p* < 0,0001) [7].

Sowohl der Immuncheckpoint-Rezeptor „programmed cell death 1“ (PD-1) als auch sein Ligand PD-L1 („programmed cell death ligand 1“), sind an der Regulation der T‑Zell-Antwort beteiligt. Während die Interaktion zwischen PD‑1 und PD-L1 im Rahmen einer physiologischen Immunantwort essenziell zur Aufrechterhaltung der Homöostase und der Vermeidung von Autoimmunität ist, dient besagte Interaktion im Tumormikromilieu als „immune escape pathway“. Eine hochregulierte PD-1- und PD-L1-Expression supprimiert eine aktive, gegen den Tumor gerichtete Immunantwort. Folglich kann eine Immuncheckpoint-Inhibition, die eine Interaktion von PD‑1 und PD-L1 unterbindet und damit eine Reaktivierung der adaptiven Immunantwort ermöglicht, zu einer kompetenten Antwort des Immunsystems gegen die Tumorzellen führen und zu einem therapeutischen Ansprechen beitragen [8]. Die Expression von PD-L1 auf Tumor- und/oder Immunzellen gehört zu den bislang am besten etablierten prädiktiven Markern für das ICI-Therapieansprechen. Eine immunhistochemische Bestimmung der PD-L1-Expression in Tumorbiopsaten wurde daher auch in den meisten der immunonkologischen Therapiestudien zu Karzinomen des Ösophagus, gastroösophagealen Übergangs oder Magens durchgeführt [8, 9].

Allerdings unterscheidet sich die PD-L1-Expression nicht nur zwischen verschiedenen Tumorentitäten, sondern unterliegt auch einer nicht unerheblichen intratumoralen Heterogenität. Sie wird darüber hinaus durch verschiedene biologische sowie auch durch methodische Faktoren beeinflusst. Gleichzeitig bietet die immunhistochemische Bestimmung der PD-L1-Expression einen hierzulande flächendeckend verfügbaren, technisch etablierten und wirtschaftlich vertretbaren Ansatz, um diejenigen Patienten zu identifizieren, die mit höherer Wahrscheinlichkeit von einer ICI-Behandlung profitieren können.

Mit dem Ziel, eine qualitätsgesicherte und standardisierte Biomarkerdiagnostik für die optimale Versorgung der Tumorpatienten zu etablieren, hat ein deutsches Expertenkonsortium auf Basis der verfügbaren Literatur und langjährigen Praxiserfahrung zu folgenden Themenbereichen konsensbasierte Empfehlungen erarbeitet:

- prädiktive Biomarker im oberen Gastrointestinaltrakt,
- Anforderungen an das Probenmaterial,
- Umgang mit diskordanten Befunden,
- qualitätsgesicherte PD-L1-Testung.

## Prädiktive Biomarker im oberen Gastrointestinaltrakt

Im Verständnis der molekularen Pathogenese von Karzinomen des Gastrointestinaltrakts wurden insbesondere in den vergangenen 10 Jahren substanzielle Fortschritte erzielt, was in der Folge veränderte Therapieregime ermöglichte – weg von einer ausschließlich an der Tumortypisierung orientierten Standard-Chemotherapie hin zu einer zusätzlich von molekularen Biomarkern gesteuerten, zielgerichteten Therapie [8, 10]. Eine wesentliche Herausforderung für die biomarkerbasierte Testung besteht unter anderem in der inter- und intratumoralen Heterogenität der Karzinome [11].

Eine vom TCGA-Konsortium (TCGA, The Cancer Genome Atlas) vorgeschlagene Subtypisierung (Epstein-Barr-Virus[EBV]-assoziierte, mikrosatelliteninstabile [MSI], genomisch stabile [GS] bzw. chromosomal instabile [CIN] Subtypen) bildet auch die molekulare Heterogenität des Magenkarzinoms ab [11, 12], ist aber für individualisierte Therapieentscheidungen derzeit noch von untergeordneter Bedeutung [13]. Dahingegen gehört die immunhistologische Bestimmung der HER2-Expression bzw. der Nachweis einer *HER2*-Genamplifikation mittels chromogener In-situ-Hybridisierung oder Fluoreszenz-in-situ-Hybridisierung (CISH/FISH) bereits zu den etablierten Biomarkern bei fortgeschrittenen/metastasierten Magenkarzinomen [2, 14, 15] sowie Adenokarzinomen des Ösophagus und des gastroösophagealen Übergangs [3, 16]. Die Angaben zur Häufigkeit einer HER2-Überexpression in Magenkarzinomen variieren zwischen 4,4 und 53,4 % (gewichtetes Mittel: 17,9 %) [17] und bei Karzinomen des gastroösophagealen Übergangs sowie Adenokarzinomen des Ösophagus zwischen 5 und 30 %. Dies ist u. a. auf heterogene intratumorale Expressionsmuster, aber auch auf die Tumorlokalisation oder unterschiedliche HER2-Scoringvorgaben zurückzuführen [18].

Neben der Erfassung des HER2-Status wird in klinischen Praxisleitlinien derzeit auch die Evaluation des PD-L1-Status empfohlen – meist anhand des CPS (Anzahl der positiven Tumorzellen und Immunzellen) beim metastasierten Magenkarzinom und/oder bei Karzinomen des Ösophagus – auch mithilfe des TPS, der den prozentualen Anteil an PD-L1-exprimierenden Tumorzellen im Verhältnis zu allen auf den jeweiligen Tumorproben vorliegenden Tumorzellen beschreibt [2, 3, 14].

Andere Charakteristika wie die MSI oder eine EBV-Positivität, die auch Teil der molekularen Subtypisierung des Magenkarzinoms sind, können bei der Prädiktion eines Ansprechens auf eine ICI-Therapie von zusätzlicher Bedeutung sein. Laut einer Metaanalyse von 4 randomisierten Studien, in denen die Rolle der MSI auf das Ansprechen auf eine ICI-Therapie beim fortgeschrittenen Magenkarzinom ausgewertet wurde, scheinen Patienten mit MSI-high(H)-Status für eine Immuntherapie besonders sensitiv zu sein [19]. Eine latente EBV-Infektion, die bei fast 9 % der Adenokarzinome des Magens angenommen wird, wird ebenfalls als ein prädiktiver Marker für das Ansprechen auf eine Therapie mit ICI diskutiert [20]. Darüber hinaus lassen sich insbesondere beim Magenkarzinom verschiedene Marker aufführen, die potenziell prognoserelevant sind, aber mehrheitlich noch keinen Eingang in die Routineanwendung bzw. die aktuellen Leitlinien gefunden haben: z. B. eine Überexpression von EGFR, c‑MET, EGF/TGF‑α, VEGF‑A oder CD44-aberranten Transkripten, eine *NTRK*-Genfusion, eine reduzierte Expression von E‑Cadherin oder die Expression bestimmter Matrixmetalloproteinasen wie MMP1, MMP7, MMP10. Die Auswahl der für die klinische Routine bereits etablierten Biomarker im Bereich des oberen Gastrointestinaltraktes bleibt damit derzeit noch vergleichsweise übersichtlich [16].

Allerdings können vor Ort zentrumsspezifisch unterschiedliche Empfehlungen und Protokolle bestehen, die individuell die Erhebung weiterer molekularer Marker vorsehen, wie z. B. den EBV-Status oder die Tumormutationslast (TMB, „tumor mutational burden“) [8, 21].

### Konsens

Die gewebebasierte Bestimmung prädiktiver (molekularer) Biomarker hat einen enormen Bedeutungsgewinn erfahren und stellt eine unabdingbare Voraussetzung für die ICI-Therapie und die sich weiter mit einer sehr hohen Dynamik entwickelnden ICI-basierten Therapiekonzepte dar. Sie leistet damit einen zentralen Beitrag für die sich daraus ergebenden signifikanten Überlebensvorteile der Patienten.

**Figure 1:**
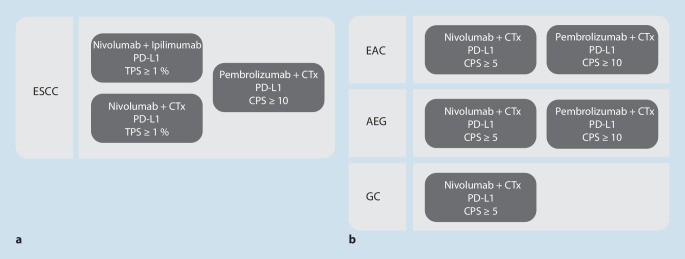

Eine vom Tumorstadium unabhängige sog. Reflextestung bei jeder Erstdiagnose wird insbesondere in frühen Tumorstadien jedoch nicht empfohlen, solange noch kurative Therapieansätze in Betracht kommen. Die prädiktive Biomarkertestung sollte aktuell vielmehr bedarfsgerecht in Abhängigkeit vom Tumorstadium, der unmittelbaren Therapierelevanz und dem aktuellen Zulassungsstatus der verfügbaren Therapieansätze erfolgen, zumal die derzeitige Therapielandschaft einschließlich der gegenwärtigen Zulassungssituation einem überaus dynamischen Wandel unterworfen ist (Abb. 1).

Zu den validierten prädiktiven Biomarkern beim Adenokarzinom gehören

- die immunhistochemische Bestimmung der Expression von PD-L1 (CPS und TPS),
- die Bestimmung der HER2-Expression (und/oder *HER2*-Amplifikation),
- die Bestimmung des MSI-Status bzw. Mismatch-Reparaturstatus (MMR) bei fortgeschrittenen Adenokarzinomen des Ösophagus (wobei MSI sehr selten ist), den Adenokarzinomen des gastroösophagealen Übergangs und des Magens [2].

Als prädiktiver Biomarker beim metastasierten oder lokal fortgeschrittenen, nicht kurativ behandelbaren Plattenepithelkarzinomen des Ösophagus validiert ist

- die immunhistochemische PD-L1-Bestimmung (TPS/CPS) [3].

## Anforderungen an das verwendete Probenmaterial

Durch eine hohe intratumorale Heterogenität bzw. Diversität der Tumorzellklone ergibt sich auch ein heterogenes Verteilungsmuster der zu analysierenden molekularen Biomarker bei gastroösophagealen Tumoren. Sowohl für gastroösophageale Adenokarzinome [22] als auch für Plattenepithelkarzinome des Ösophagus [23] ergeben sich in Bezug auf die PD-L1-Expression Hinweise auf eine nicht unerhebliche intratumorale Heterogenität, wobei Unterschiede zwischen Primarius und etwaigen Lymphknoten- und Fernmetastasen existieren [20, 23]. Die Tumordiagnostik und Biomarkerbestimmung erfolgen im palliativen Kontext häufig an aufgrund der beschränkten Anzahl der erhältlichen Proben limitiertem Biopsiematerial. Das oft nur wenige Millimeter große Gewebe repräsentiert nur einen Ausschnitt der Gesamttumormasse und birgt die Möglichkeit eines Stichprobenfehlers.

Die Aufarbeitung von 465 Tumorresektaten therapienaiver Patienten mit Karzinomen des Magens oder gastroösophagealen Übergangs an der Kieler Universitätspathologie hat gezeigt, dass sich PD-L1-positive Tumorzellen in 33,1 % der Fälle durch oberflächliche Biopsate des Primärtumors (< 2,5 mm von der Tumoroberfläche entfernt) nicht erfassen ließen, sondern diese nur im Tumorzentrum oder nahe der Invasionsfront nachweisbar waren [24]. Zum Erreichen einer möglichst hohen Konkordanz der Ergebnisse, die durch die Beurteilung von Biopsien und an größeren Schnittpräparaten von Resektaten erhoben werden, wurde in einer asiatischen Studie die Mindestanzahl von 5 zentralen Tumorbiopsien ermittelt (bei einem Cut-off ≥ 1 % für die PD-L1-Positivität). Bei weniger als 5 Biopsien ließ sich den Autoren zufolge keine hinreichende Sensitivität und Konsistenz erreichen. Wenn die PD-L1-Expression im nichtresezierbaren Tumorstadium alleinig in Biopsaten bestimmt werden muss, können jedoch auch mehr als 5 Biopsien erforderlich sein [25]. In den aktualisierten Praxisleitlinien der ESMO werden „multiple endoskopische Biopsien“, d. h. konkret 5–8 Biopsien beim Magenkarzinom bzw. 6 oder mehr als 6 Biopsien beim Ösophaguskarzinom empfohlen, damit für die histologische und molekulare Analyse suffizientes Ausgangsmaterial zur Verfügung steht [2, 3]. Die deutsche S3-Leitlinie zur Diagnostik und Therapie des Magenkarzinoms empfiehlt bei Verdacht auf ein Magenkarzinom grundsätzlich die Entnahme von mindestens 8 Biopsien aus allen suspekten Arealen, wobei es weniger auf die absolute Anzahl als auf die Anzahl der tumortragenden Biopsien ankommt [26]. Da es sich bei PD-L1 um einen dynamischen Biomarker handelt, dessen Expression sich im Verlauf der Erkrankung und auch in Abhängigkeit von den durchgeführten Therapien ändern kann, ist auch die Wahl der Probe im zeitlichen Kontext der Erkrankung entscheidend [27–29].

In einer weiteren Patientenkohorte aus Kiel wurde bei neoadjuvant behandelten Karzinomen des Magens und des gastroösophagealen Übergangs (*n* = 141) eine veränderte PD-L1-Expression unter einer neoadjuvanten Chemotherapie beobachtet. Bei insgesamt geringerer PD-L1-Expression wiesen insbesondere Patienten mit schlechtem Ansprechen auf die neoadjuvante Chemotherapie gegenüber der therapienaiven Kohorte [24] eine vermehrte Expression von PD-L1 auf (sowie PD‑1 und VISTA [„V-domain Ig suppressor of T cell activation“]; [30]). Vor diesem Hintergrund erscheint es sinnvoll, die PD-L1-Testung insbesondere bei initial negativem PD-L1-Status sequenziell im zeitlichen Verlauf der Tumorerkrankung und insbesondere bei Progression erneut durchzuführen. Das dann zur Beurteilung verwendete Material sollte aus einem Resektat oder Rebiopsien (auch aus einer Metastase) stammen [24]. Die bislang einzige Metaanalyse, die sich mit der Konversion eines Biomarkerstatus zwischen Primärtumoren und gepaarten Metastasen befasst hat, umfasste bei der PD-L1-Konversion 38 Studien über verschiedene Tumorentitäten hinweg. Ermittelt wurde eine gepoolte Diskordanzrate von 22 %. Dabei wurden Konversionen vom positiven zum negativen PD-L1-Expressionsstatus häufiger beobachtet (41 %) als umgekehrt (16 %) [31]. Diskordante Befunde zwischen Primärtumor und korrespondierender Metastase oder zwischen Biopsie und Tumorresektat [27] sind auch beim HER2-Expressionsstatus bekannt (Diskordanzrate von 9–16 % für HER2) [32].

Grundsätzlich liegt bei Merkmalen, die eine intratumorale Heterogenität aufweisen, und bei Biomarkern, die an Biopsaten analysiert werden, ein Risiko für einen Stichprobenfehler vor. Ein erhöhtes Risiko für ein nichtrepräsentatives, insbesondere falsch negatives Testergebnis besteht bei einem Drittel der als PD-L1-positiv getesteten Magenkarzinome [24]. Das deutsche Expertenkonsortium spricht sich dafür aus, zur Reduzierung dieses Risikos

- beim Resektionsmaterial einen repräsentativen Schnitt mit tumorassoziiertem Stroma zu erreichen,
- die Entnahme von mindestens 5 (optimal 6–8) tumortragenden endoskopischen Biopsien für die derzeit bei Karzinomen des oberen Gastrointestinaltraktes erforderliche Biomarkerdiagnostik anzustreben,
- die Entnahme der Biopsate aus verschiedenen, zufällig gewählten Arealen des erreichbaren Tumormaterials durchzuführen.

Das endoskopisch gewonnene Biopsat dient bei fortgeschrittener Erkrankung und damit häufig palliativer Therapiesituation der Primärdiagnose und stellt in dieser Situation auch häufig das einzig verfügbare Gewebe dar, an dem die Bestimmung der Biomarker erfolgt. In Einzelfällen sollte jedoch im Verlauf der Erkrankung und bei Verfügbarkeit weiteren Materials, beispielsweise aus einem Resektat oder auch Metastasengewebe, eine Reevaluation des Biomarkerstatus erfolgen, da sich die Expression von PD-L1 und von HER2 sowohl im Verlauf der Erkrankung als auch durch erfolgte Therapien verändern kann.

Verbindliche Empfehlungen, wann konkret eine Rebiopsie bzw. Retestung erfolgen sollte, können derzeit evidenzbasiert nicht einheitlich formuliert werden und bedürfen weiterer Studien. Aufgrund der hohen Relevanz für die Therapieentscheidung erscheint es nach übereinstimmenden Erfahrungen des deutschen Expertenpanels jedoch derzeit sinnvoll, die Biomarkerbestimmung

- bei biomarkernegativer Vorbiopsie und chemotherapeutisch neoadjuvant vorbehandelten Fällen an einer Rebiopsie zu wiederholen und dazu auf das biologisch aktuellste Tumormaterial bzw. bei fehlender Zugänglichkeit auf das verfügbare aktuellste Archivmaterial zurückzugreifen,
- im Falle eines Rezidivs an einer Rebiopsie zu wiederholen, wenn der Tumor vorbehandelt wurde bzw. das vorherige Testergebnis beim Primärtumor negativ war.

Nach derzeitiger Datenlage kann die Therapieentscheidung nicht allein anhand der Biomarker getroffen werden, sondern bedarf der interdisziplinären Zusammenschau und Einordnung aller vorliegenden Informationen und Befunde der Patienten.

## Anmerkungen zum Umgang mit diskordanten Befunden

Die PD-L1-Expression ist neben einer Dynamik insbesondere auch durch eine hohe intratumorale Heterogenität gekennzeichnet, was das Risiko für Stichprobenfehler, insbesondere in Biopsiematerial, birgt. Erschwerend kommt hinzu, dass ein positiver PD-L1-Status nicht zwingend ein gutes Ansprechen der Patienten auf eine Tumortherapie vorhersagt. PD-L1-negative Tumoren (darunter ggf. auch falsch negative Befunde infolge von Tumorheterogenität oder Fälle mit diskordantem Expressionsmuster zwischen Primarius und Metastase) können auf die ICI-basierte Behandlung ansprechen bzw. können positiv getestete Tumoren kein Ansprechen aufweisen. Darüber hinaus wurden in klinischen Studien von verschiedenen Herstellern in den unterschiedlichen Tumorentitäten eigene Scoringalgorithmen und Cut-off-Werte ermittelt und etabliert, was eine sichere Vergleichbarkeit ebenfalls erschweren kann.

Festzuhalten ist, dass die Ergebnissicherheit mit zunehmender Anzahl der verfügbaren tumortragenden Biopsien steigt und das Risiko eines Stichprobenfehlers und damit für falsch negative Ergebnisse abnimmt, was sich auch in der Empfehlung zur Anzahl der zu analysierenden Proben niederschlägt [9, 25].

### Konsens

Die Therapieentscheidung und Einordnung der Ergebnisse der Biomarkertestung obliegt dem behandelnden Kliniker. Insbesondere bei einer Progression der Erkrankung, unterschiedlichen Ergebnissen der Biomarkertestung im Verlauf oder aber vorangegangener Therapie bedarf es von klinischer Seite einer individuellen Abwägung zur Indikationsstellung einer (Re‑)Biopsie bzw. einer erneuten Testung. Die pathologischen Befunde liefern für die jeweilige Therapieentscheidung wertvolle Informationen, sodass aus Sicht des deutschen Expertenpanels folgende Angaben strukturiert dokumentiert und übermittelt werden sollten:

- ausgewertetes Material (aktuelles Material/Archivmaterial, Tumorresektat/(Re‑)Biopsie, Primärtumor/Metastase),
- Angabe über die zur Verfügung stehende Anzahl der tumortragenden Proben,
- ggf. Faktoren hervorheben, die die Repräsentativität des pathologischen Befundes einschränken,
- verwendeter Primärantikörper (Klon) sowie die verwendete Plattform bzw. der verwendete Färbeautomat für die Nachvollziehbarkeit durch Kollegen,
- Scoring und Ergebnisse als absolute und konkrete Zahlenwerte (CPS und TPS) unabhängig von der vorliegenden Tumorentität und ggf. Angaben zu diskordanten Befunden.

Für eine möglichst sichere Diagnosestellung sollten von klinischer Seite nach Möglichkeit Angaben zur Vorgeschichte (v. a. eine etwaig erfolgte Vortherapie) und eine Therapieplanung (z. B. „ICI-Therapie geplant“) zur Verfügung gestellt werden.

## Hinweise für eine qualitätsgesicherte PD-L1-Bestimmung

Auf dem Markt ist eine Vielzahl an diagnostischen Antikörpern und Assays für die PD-L1-Immunhistochemie verfügbar. Auch wenn klinische Studien mit je nach Hersteller und Entität bestimmten Antikörperklonen bzw. Assays und Kits durchgeführt wurden, ergibt sich daraus in Deutschland keine zwingende Notwendigkeit, diese auch im klinischen Alltag auszuwählen. Für Pathologen in Deutschland besteht Freiheit bei der diagnostischen/medizinischen Methodenwahl und damit auch bei der Auswahl des Testsystems. Damit unterscheidet sich die Situation hierzulande grundsätzlich von der Testungssituation beispielsweise in den USA, wo durch FDA-definierte „companion diagnostics“ die Verwendung von bestimmten Antikörpertests im Zusammenhang mit einer klinischen Fragestellung und der Zulassung eines Medikaments festgelegt sind. Eine prinzipielle Vergleichbarkeit und vor allem Reproduzierbarkeit der Ergebnisse der verschiedenen, in klinischen Studien verwendeten und validierten Antikörper und Plattformen ist gegeben, wofür auch die Ergebnisse einer der ersten publizierten Harmonisierungsstudien beim Magenkarzinom sprechen (22C3, SP263) [33]. Die Interpretation der immunhistochemischen PD-L1-Untersuchungen erfordert vom jeweiligen Pathologen infolge der Komplexität der Testung fundierte Kenntnisse und ein entsprechendes Training für die entsprechenden Scores und Cut-offs der verschiedenen Entitäten (Deutsche Akkreditierungsstelle, DAkkS) [34]. Von vorrangiger Bedeutung ist daher die Etablierung einer standardisierten, reproduzierbaren Immunhistochemie, die u. a. durch die regelmäßige Teilnahme an externen Ringversuchen (z. B. bereitgestellt durch die Qualitätssicherungs-Initiative „Pathologie QuIP GmbH“) und die Akkreditierung sowie auf Nicht-Ringversuch-bezogene Qualitätssicherungsmaßnahmen (z. B. NordicQC) zur kontinuierlichen Erhaltung und Verbesserung von Qualität sichergestellt wird. Eine umfassende Qualitätssicherung der molekularpathologischen Diagnostik ist Bestandteil einer hochwertigen klinischen Krebsversorgung [35]. Eine Teilnahme der befundenden Pathologen an entsprechenden Trainings stellt einen zusätzlichen Baustein dar, um eine hohe Ergebnisqualität sicherzustellen.

### Konsens

Bei der Auswahl der Testsysteme der PD-L1-Immunhistochemie besteht in Deutschland Methodenfreiheit. Für die präanalytische Prozessierung sei auf die Vorgaben der jeweiligen Testanbieter verwiesen. Wichtig sind die Schulung der an der Auswertung beteiligten Pathologen und die regelmäßige Teilnahme an externen Ringversuchen zur PD-L1-Immunhistochemie. Für die Bearbeitungszeit zwischen Materialeingang und den fertigen und übermittelten pathologischen Befunden gibt es bisher keine einheitlichen oder verbindlichen Vorgaben, wobei trotz der geschilderten Komplexität der pathologischen Diagnostik der Befund in der Regel innerhalb von 3–5 Arbeitstagen abgeschlossen sein sollte.
